# IFNγ+ Treg in-vivo and in-vitro represent both activated nTreg and peripherally induced aTreg and remain phenotypically stable in-vitro after removal of the stimulus

**DOI:** 10.1186/s12865-015-0111-2

**Published:** 2015-08-13

**Authors:** Volker Daniel, Karina Trojan, Martina Adamek, Gerhard Opelz

**Affiliations:** Department of Transplantation-Immunology, Institute of Immunology, University Hospital Heidelberg, Im Neuenheimer Feld 305, 69120 Heidelberg, Germany

**Keywords:** IFNγ+ nTreg, IFNγ+ aTreg, Foxp3 TSDR demethylation, IFNγ, Th1, Healthy individuals

## Abstract

**Background:**

IFNγ-producing CD4+CD25+Foxp3+CD127- Treg represent the first line of Treg during an immune response. In the present study we determined whether IFNγ+ Treg in-vivo and in-vitro are Helios-positive representing activated natural (nTreg) or Helios-negative representing adaptive Treg (aTreg) and whether they originate from CD4+CD25+ and/or CD4+CD25- PBL. Furtheron, we investigated whether they are inducible by recombinant IFNγ (rIFNγ) as a single stimulus, decrease in-vitro after elimination of the stimulus, and have a demethylated Foxp3 Treg-specific demethylated region (TSDR) which is associated with stable Foxp3 expression.

**Method:**

Subsets of IFNγ+ Treg were determined in peripheral blood of healthy controls using eight-color flow cytometry and were further investigated in-vitro. Foxp3 TSDR methylation status was determined using bisulphite polymerase chain reaction (PCR) and high resolution melt (HRM) analysis.

**Results:**

Nearly all Treg in the peripheral blood were Helios+IFNγ- (1.9 ± 1.1/μl) and only few were Helios+IFNγ+ or Helios-IFNγ+ Treg (both 0.1 ± 0.1/μl). Enriched IFNγ+ Treg subsets showed in part strong Foxp3 TSDR demethylation. In-vitro, rIFNγ was unable to induce Treg. CD4+CD25+ enriched PBL stimulated with PMA/Ionomycin in the presence of rIFNγ were rather resistant to the effect of rIFNγ, in contrast to CD4+CD25- enriched PBL which showed increasing total Treg with Helios+ Treg switching from IFNγ- to IFNγ+ and increasing Helios-IFNγ+ Treg. The data indicate that rIFNγ, in combination with a polyclonal stimulus, activates nTreg and induces aTreg. When phorbol 12-myristate 13-acetate (PMA)/Ionomycin was washed out from the cell culture after 6 h stimulation, Treg induction continued for at least 96 h of cell culture, contradicting the hypothesis that removal of the stimulus results in significant decrease of IFNγ- and IFNγ+ CD4+CD25+Foxp3+CD127- Treg due to loss of Foxp3 expression.

**Conclusions:**

IFNγ+Helios- aTreg as well as IFNγ+Helios+ nTreg are detectable in the blood of healthy individuals, show in part strong Foxp3 TSDR demethylation and are inducible in-vitro. The present data provide further insight concerning the in-vivo and in-vitro characteristics of IFNγ+ Treg and help to understand their role in immunoregulation. Alloantigen-specific demethylated IFNγ+Helios+ nTreg might represent a suitable marker for monitoring graft-specific immunosuppression in renal transplant recipients.

## Background

T regulator cells (Treg) in the peripheral circulation of humans are usually IFNγ-. However, during stimulation CD4+CD25+CD127-Foxp3+ Treg are formed that co-express IFNγ, as reviewed by Daniel et al. [1]. IFNγ+ Treg are detectable in the blood of renal transplant recipients with good long-term graft function and in patients with autoimmune disease, such as type-1 diabetes and multiple sclerosis [2–4]. They co-express IFNγ receptors on the cell surface and are inducible by IFNγ (auto- and paracrine activation) [5, 6]. In addition to IFNγ, the cells produce TGFß and/or IL10 and co-express cell surface receptors that are involved in cell-cell contact inhibition as well as effector cell killing, such as CD152, CD178, CD95, and CD279 [6, 7]. Separated CD4+CD25+CD127-IFNγ+ Treg suppress MLCs unspecifically, although the strongest suppression is observed in antigen-specific mixed lymphocyte culture (MLC) settings [5, 7, 8]. The stronger the HLA incompatibility and proliferation in MLC experiments, the stronger the induction of CD4+CD25+Foxp3+IFNγ+ PBL [8]. Interestingly, patients with poor long-term allograft function after renal transplantation are able to form CD4+CD25+Foxp3+IFNγ+ PBL in MLC with pretransplant obtained peripheral blood lymphocytes (PBL) [8]. However, posttransplant these patients showed lower frequencies of these cells in the periphery than renal transplant recipients with good long-term graft outcome [2]. This finding suggests that patients with poor long-term graft outcome did not develop or lost this particular Treg subset posttransplant, perhaps because of intensified immunosuppressive treatment. The in-vitro behavior of IFNγ+CD4+CD25+Foxp3+CD127- Treg suggests that they form the first line of Treg, patrolling in the body and searching for IFNγ and initial immune responses that they subsequently suppress.

Origin and stability of suppressive function of IFNγ+ Treg are important factors with respect to the clinical relevance of this Treg subset [1]. Usually, the Foxp3 Treg-specific demethylated region (TSDR) of Tregs is demethylated, whereas that of conventional T cells is methylated [9, 10]. TSDR methylation persists during transient expression of Foxp3 by convential T cells or unstable TGFß-induced Treg, whereas drug-induced demethylation of conventional T cells results in stable Foxp3 expression, suggesting that lineage stability in Treg may be epigenetically regulated. Signaling through IFNγR and IL12R, in combination with T cell receptor (TCR) engagement, induces strong expression of the transcription factor T-bet, which drives the differentiation of conventional T cells to a T helper type 1 (Th1) lineage [11]. The Ikaros family transcription factor Helios has been shown to be selectively expressed in nTreg of thymic origin but not in peripherally induced aTreg [12]. McClymont et al. reported that the majority of in-vitro induced IFNγ+ Tregs did not express Helios, suggesting that they were generated extra-thymically [4]. Alternatively, they might belong to a minority of Helios- nTreg with demethylated TSDR expressing Foxp3, CD39, CTLA-4, CCL3 and IFNγ, as published by Himmel et al. [13]. Further experiments showed that nTreg can polarize towards IFNγ+ T cells in-vitro by IL12 conditioning whereby they remain Helios+, suggesting that part of the thymic-derived Treg population exhibits plasticity in cytokine production and expresses a Th1-like phenotype [4]. Hall et al. reported that rat nTreg stimulated with antigen and IFNγ or IL12 expand and differentiate to antigen-specific IFNγ+ nTreg that have a 100- to 1000-fold increased suppressor potency as compared to nTreg [14, 15]. This strongly potent antigen-specific nTreg subset with a Th1-like phenotype might represent a suitable marker for monitoring graft-specific immunosuppression in renal transplant recipients.

In the present study we examined the origin of IFNγ+ Treg as determined by Helios positivity in peripheral blood and PMA/Ionomycin-stimulated cell cultures performed with PBL from healthy individuals. Furtheron, we investigated whether IFNγ+ Treg can be induced by recombinant IFNγ (rIFNγ) without additional stimulus and whether IFNγ+ Treg after removal of the stimulus remain IFNγ+Foxp3+ or, alternatively, decrease and differentiate to IFNγ+Foxp3- Th1 and/or IFNγ-Foxp3+ Treg. Based on findings in mouse experiments, Feng et al. reported that in-vitro induced IFNγ+ Treg suppress inflammation effectively when transferred to mice with colitis but differentiate to Th1 lymphocytes when adoptively transferred to mice without inflammation [16]. Finally, we determined the Foxp3 TSDR demethylation status of separated IFNγ+ Treg in order to assess stability of Foxp3 expression.

## Results

### Treg subsets in peripheral blood of healthy controls

**Fig. 1:**
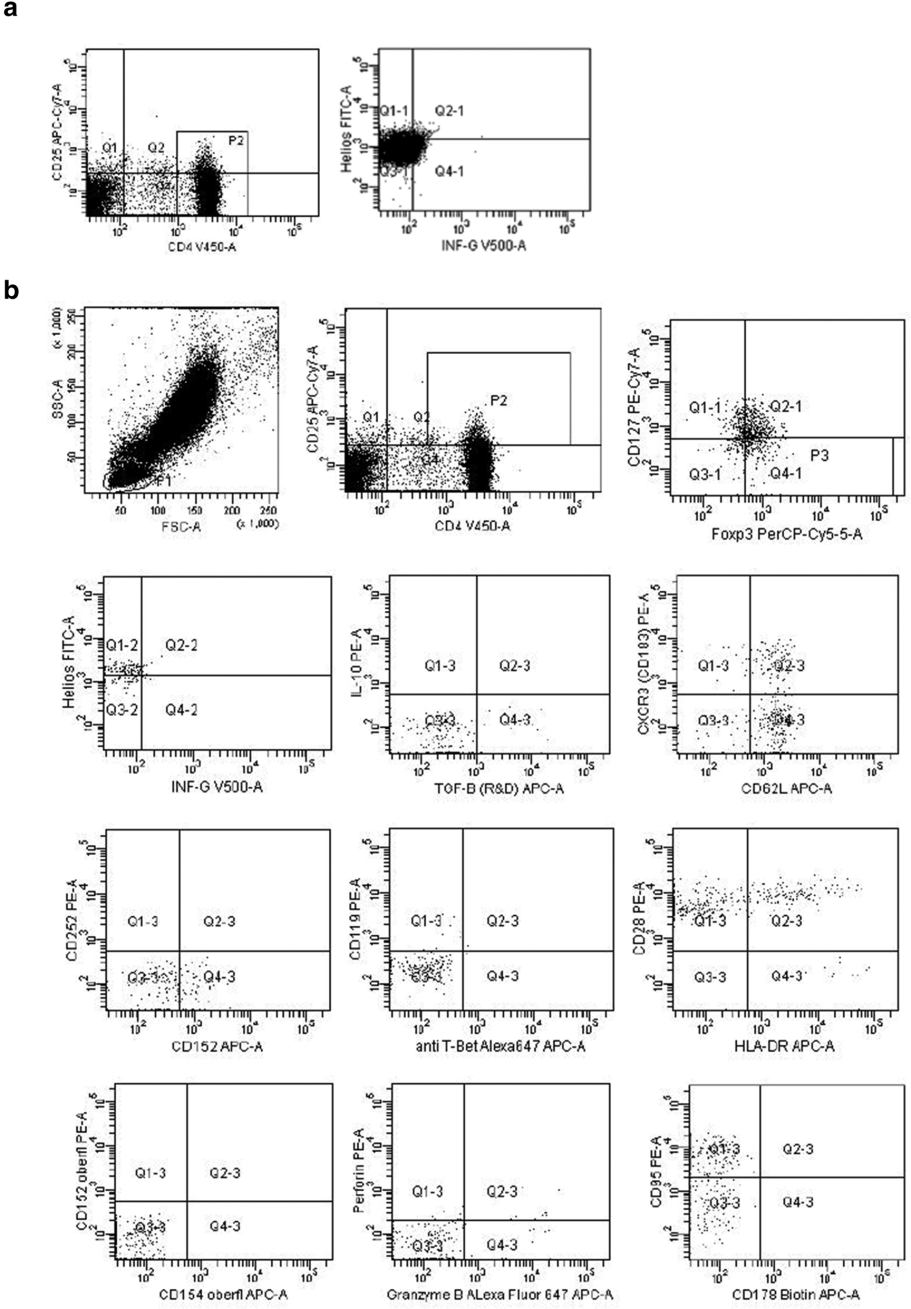
Determination of Treg subsets. **a** Total CD4+ lymphocytes of a healthy individual were gated (gate P2) and further analysed concerning intracellular Helios and IFNγ positivity. The majority of circulating CD4+ lymphocytes was Helios- and this gate was used for all further flow cytometric analyses of Helios positivity. **b** Stepwise gating strategy for Treg subset determination: first, lymphocytes gate (P1), then CD4+CD25+ PBL gate (P2), Foxp3+CD127- gate (P3), and finally Helios/IFNγ gate. Further CD4+CD25+Foxp3+CD127- Treg subsets (based on gate P3) were analysed using the depicted gate settings for IL10/TGFß, CXCR3/CD62L, CD252/CD152 (CD152 intracellular), CD119/Tbet, CD28/HLA-DR, CD152/CD154 (both surface), Perforin/GranzymeB, and CD95/CD178

**Fig. 2:**
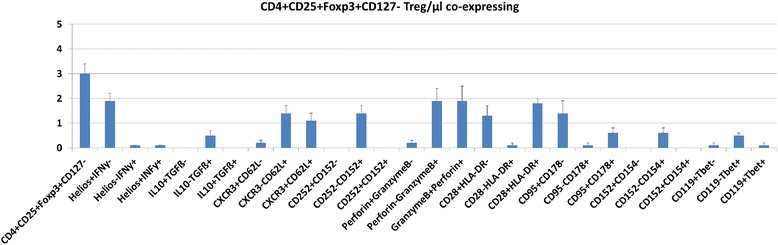
Absolute counts of different CD4+CD25+Foxp3+CD127- Treg subsets in the blood of 12 healthy controls. Two-thirds of all CD4+CD25+Foxp3+CD127- Treg were Helios+IFNγ- nTreg, only a small minority represents Helios+IFNγ+ activated nTreg or Helios-IFNγ+ peripherally induced aTreg. The additionally investigated parameters give insight into the immunosuppressive armamentarium and the mechanisms of IFNγ+ Treg function. Data are given as mean ± SEM

Figure 1 demonstrates the gating strategy. Figure 2 depicts Treg subset numbers in the blood of 12 healthy individuals. Mean ± SEM (range) of circulating CD4+ PBL was 799 ± 220/μl (483–1179/μl) and of circulating CD4+CD25+Foxp3+CD127- Treg 3.0 ± 1.4/μl (1.2–6.4/μl). Approximately two-thirds of all Treg expressed the classical nTreg phenotype Helios+IFNγ- (1.9 ± 1.1/μl; 0.7–3.7/μl). In contrast, only a small minority of Treg produced IFNγ and was Helios+IFNγ+ (0.1 ± 0.1/μl; 0–0.4/μl) or Helios-IFNγ+ (0.1 ± 0.1/μl; 0–0.5/μl). Half of IFNγ+ Treg appear to originate from the thymus (Helios+) and represent activated nTreg, whereas the other half appear to be induced in the periphery (Helios-) and represent aTreg. CD4+CD25+Foxp3+CD127- Treg were found to express TGFß, CD62L, CXCR3, CD152, Perforin, Granzyme B, CD28, HLA-DR, CD95, CD178, CD154, Tbet and IFNγR1 CD119. The data suggest that IFNγ+ Treg have the potential to enter secondary lymphoid organs as well as inflamed tissues (CD62L, CXCR3) and are able to induce suppression of immune responses either by apoptosis (CD95, CD178), cell death (Perforin, Granzyme B), cytokine secretion (TGFß) or cell-cell interaction (CD152, CD154). Moreover, they have the potency to regulate production as well as consumption of IFNγ in the cell by surface receptors (CD119) and induction of transcription factors (Tbet). Interestingly, IL10+ Treg phenotypes were undetectable in the circulation whereas TGFß+ IFNγ+ Treg were rather frequent (Fig. 2).

### Cell cultures stimulated with rIFNγ only

**Fig. 3:**
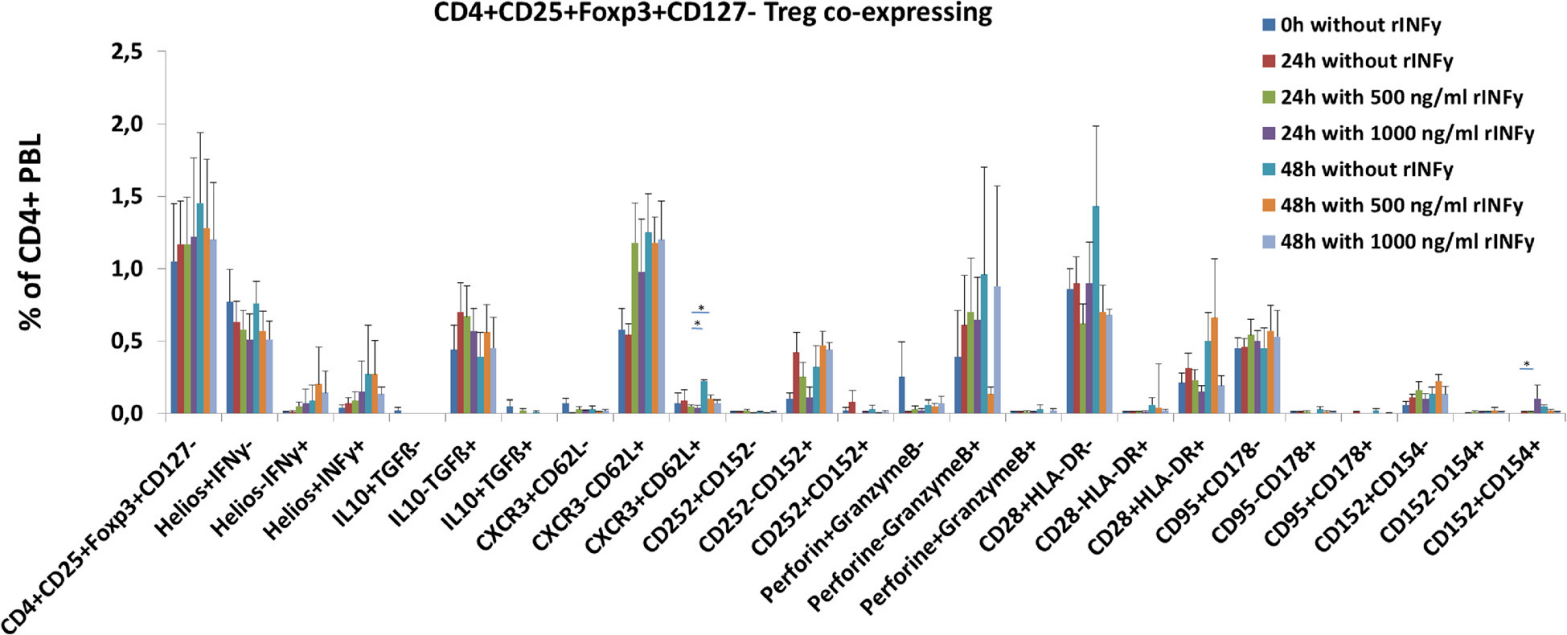
Cell cultures stimulated with rIFNγ only. Unseparated PBL of 4 healthy individuals were cultured for 48 h in the presence of 500 ng/ml, 1000 ng/ml or without rIFNγ. There was no significant increase of IFNγ+CD4+CD25+Foxp3+CD127- Treg during the culture period (24 h resp. 48 h: 0 ng/ml vs 500 ng/ml resp. 1000 ng/ml, *p* = n.s.). The data suggest that rIFNγ is unable to induce Treg, especially IFNγ+CD4+CD25+Foxp3+CD127- Treg, without an additional stimulus. Rather, there was a decrease of CXCR3+CD62L+ (48 h: 0 ng/ml vs 500 ng/ml resp. 1000 ng/ml rIFNγ, both *p* = 0.029) as well as CD152+CD154+ (48 h: 0 ng/ml vs 1000 ng/ml rIFNγ, *p* = 0.029) Treg. Data are given as mean ± SEM. *p ≤ 0.05; **p ≤ 0.01; ***p ≤ 0.001

As shown previously, IFNγ+ Treg express IFNγ receptors [6]. We hypothesized that IFNγ+ Treg might be inducible by recombinant rIFNγ without additional stimulus. When unseparated PBL of 4 healthy volunteers were stimulated with 500 or 1000 ng/ml rIFNγ for 24 and 48 h, there was no statistically significant increase in any of the Treg subsets depicted in Fig. 3 (24 h resp. 48 h: 0 ng/ml vs 500 ng/ml resp. 1000 ng/ml rIFNγ, *p* = n.s.). Rather, there was a decrease of CXCR3+CD62L+ (48 h: 0 ng/ml vs 500 ng/ml resp. 1000 ng/ml rIFNγ, both *p* = 0.029) as well as CD152+CD154+ (48 h: 0 ng/ml vs 1000 ng/ml rIFNγ, *p* = 0.029) Treg. The data suggest that rIFNγ is unable to induce Treg, especially IFNγ+ Treg, in the absence of an additional stimulus.

### IFNγ+ Treg induced in-vitro in the presence of both rIFNγ and PMA/Ionomycin

We speculated that rIFNγ might induce IFNγ+ Treg only in combination with an additional stimulus, such as PMA/Ionomycin, and that activated nTreg with Helios+IFNγ+ phenotype originate mainly from the CD4+CD25+ PBL preparation. We therefore investigated the induction of IFNγ+ Treg in cell cultures with unseparated PBL, enriched CD4+CD25+ and enriched CD4+CD25- PBL preparations. CD4+ PBL were enriched to 99 ± 0.6 % purity. CD4+CD25+ enriched PBL fractions contained 87 ± 9.9 % and the remaining CD4+CD25- PBL preparation 33 ± 10.6 % CD4+CD25+ PBL. PBL preparations of 5 healthy individuals were stimulated for 72 h with PMA/Ionomycin in the presence of 500 ng/ml, 1000 ng/ml, 5000 ng/ml or without rIFNγ.

#### Unseparated PBL

**Fig. 4:**
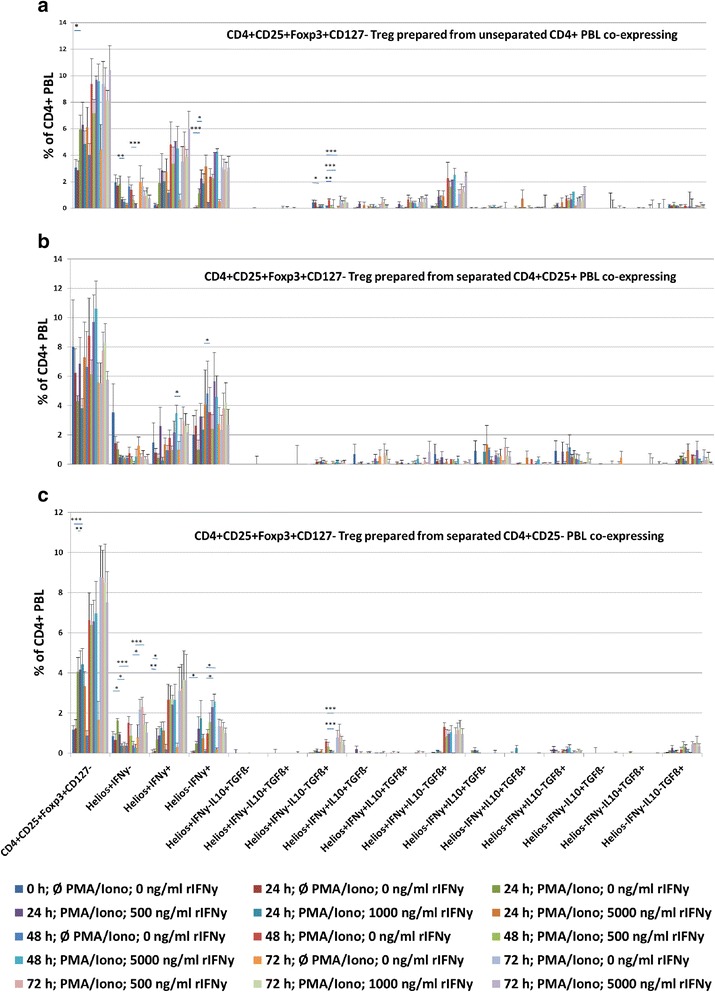
IFNγ+ Treg induced in-vitro in the presence of both rIFNγ and PMA/Ionomycin. **a** PBL of 5 healthy individuals were stimulated for 72 h using PMA/Ionomycin in the presence of 500, 1000, 5000 ng/ml or without rIFNγ. PMA/Ionomycin-stimulated cultures without rIFNγ were compared with those stimulated in the presence of rIFNγ. **b**, **c** The same assay was performed using cell cultures with enriched CD4+CD25+ or enriched CD4+CD25- PBL. The data suggest that CD4+CD25+ PBL are rather resistant to the effect of rIFNγ. In contrast, CD4+CD25- enriched PBL showed increasing total Treg with both Helios+ Treg switching from IFNγ- to IFNγ+ and increasing IFNγ+Helios- Treg indicating that rIFNγ in combination with a polyclonal stimulus induces activation of nTreg as well as induction of aTreg. Data are given as mean ± SEM. *p ≤ 0.05; **p ≤ 0.01; ***p ≤ 0.001

Stimulation of unseparated PBL with PMA/Ionomycin alone for 24 h increased the proportions of total CD4+CD25+Foxp3+CD127- (24 h: with vs without PMA/Iono: *p* = 0.056) and Helios-IFNγ+ (*p* = 0.008) Treg, but decreased the proportion of Helios+IFNγ-IL10-TGFß+ (*p* = 0.056) Treg (Fig. 4a). Addition of rIFNγ to PMA/Ionomycin-stimulated cell cultures decreased the proportions of Helios+IFNγ- (24 h resp. 48 h: PMA/Iono vs PMA/Iono + 5000 ng/ml rIFNγ, *p* = 0.016 resp. *p* = 0.008) and Helios+IFNγ-IL10-TGFß+ (48 h: PMA/Iono vs PMA/Iono + 500 resp. 1000 resp. 5000 ng/ml rIFNγ, *p* = 0.016 resp. *p* = 0.008 resp. *p* = 0.008) Treg and increased the proportion of Helios-IFNγ+ (24 h: PMA/Iono vs PMA/Iono + 5000 ng/ml rIFNγ, *p* = 0.032) Treg. It appears that rIFNγ decreased the Helios+IFNγ- and increased the Helios-IFNγ+ Treg subset in PMA/Ionomycin-stimulated cell cultures.

#### Enriched CD4+CD25+ PBL

When, instead of unseparated PBL, cell preparations enriched for CD4+CD25+ PBL were stimulated, addition of rIFNγ increased Helios-IFNγ+ (24 h: PMA/Iono vs PMA/Iono + 500 ng/ml rIFNγ, *p* = 0.056) Treg. Helios+IFNγ+ Treg decreased (48 h: PMA/Iono vs PMA/Iono + 500 ng/ml rIFNγ; *p* = 0.056) but tended to increase in the presence of higher rIFNγ concentrations in the culture medium (*p* = n.s.) (Fig. 4b).

#### Enriched CD4+CD25- PBL

Stimulation of enriched CD4+CD25- PBL with PMA/Ionomycin alone increased total CD4+CD25+Foxp3+CD127- (24 h: with vs without PMA/Iono: *p* = 0.016) and Helios+IFNγ- (*p* = 0.056) as well as Helios+IFNγ+ (*p* = 0.056) Treg (Fig. 4c). When rIFNγ was added to PMA/Ionomycin-stimulated cell cultures, a decrease of Helios+IFNγ- Treg was noted (24 h: PMA/Iono vs PMA/Iono + 1000 resp. 5000 ng/ml rIFNγ, *p* = 0.056 resp. *p* = 0.008; 48 h: PMA/Iono vs PMA/Iono + 1000 resp. 5000 ng/ml rIFNγ, *p* = 0.032 resp. *p* = 0.008) as well as of Helios+IFNγ-IL10-TGFß+ (48 h: PMA/Iono vs PMA/Iono + 1000 resp. 5000 ng/ml rIFNγ, *p* = 0.008 resp. *p* = 0.008), whereas Helios-IFNγ+ (48 h: PMA/Iono vs PMA/Iono + 1000 resp. 5000 ng/ml rIFNγ, *p* = 0.032 resp. *p* = 0.032) Treg increased. A strong increase of total CD4+CD25+Foxp3+CD127- (*p* = 0.008) and Helios+IFNγ+ (*p* = 0.016) Treg as well as an increase of Helios-IFNγ+ (*p* = 0.056) Treg was observed when freshly separated CD4+CD25- PBL were compared with CD4+CD25- PBL after 24 h stimulation with PMA/Ionomycin.

The data suggest that rIFNγ in combination with PMA/Ionomycin decreases Helios+IFNγ- and increases Helios-IFNγ+ Treg in-vitro and that this effect was more pronounced in cell cultures with enriched CD4+CD25- than in cell cultures with CD4+CD25+ PBL. Treatment with rIFNγ appears to increase total Treg that are Helios-IFNγ+ and induces a switch of Helios+ Treg from IFNγ- to IFNγ+. We conclude that rIFNγ in combination with a polyclonal stimulus induces aTreg and activates nTreg.

### Kinetics of Treg subsets in-vitro in culture of CD4+CD25+ or CD4+CD25- PBL preparations after elimination of the stimulus

**Fig. 5:**
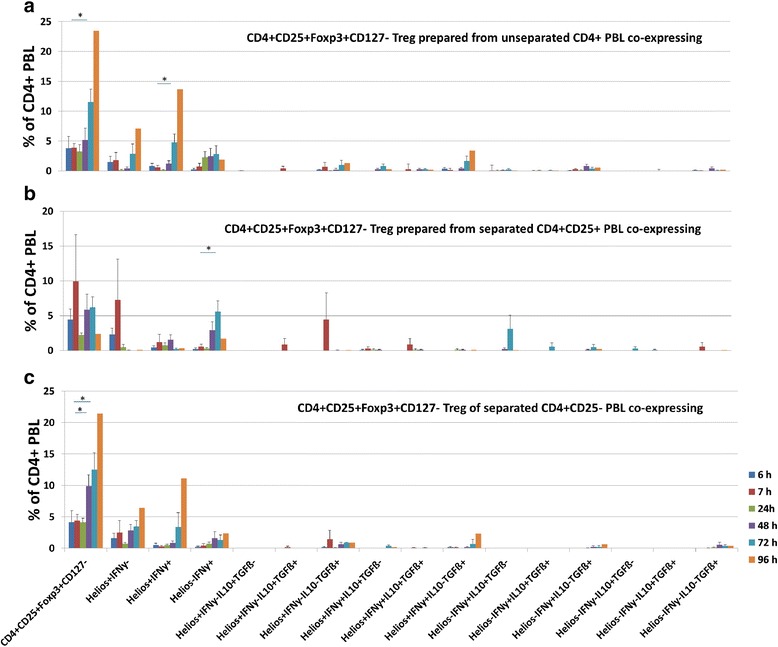
Kinetics of Treg subsets induced in-vitro from CD4+CD25+ or CD4+CD25- PBL preparations after elimination of the stimulus. Unseparated as well as enriched CD4+CD25+ and CD4+CD25- cell preparations were stimulated with PMA/Ionomycin for 6 h and Treg subsets were determined. Mitogen was removed by several washes of the cell culture and Treg subsets were determined again immediately after the washes at 7 h and at 24, 48, 72 and 96 h after initiation of the cell culture. Assays were performed with PBL from 3 healthy volunteers. The measurement 96 h after initiation of the cell culture was performed with only one cell donor. CD4+CD25+Foxp3+CD127- Treg increased after removal of the stimulus. The strong increase of CD4+CD25+Foxp3+CD127- Treg was also observed when instead of unseparated PBL CD4+CD25- enriched PBL fractions were used. In contrast, CD4+CD25+ enriched PBL fractions showed only an increase of Helios-IFNγ+ Treg. Our data do not support the hypothesis that removal of the stimulus results in a significant decrease of IFNγ- as well as IFNγ+ CD4+CD25+Foxp3+CD127- Treg due to loss of Foxp3 expression. Data are given as mean ± SEM. *p ≤ 0.05; **p ≤ 0.01; ***p ≤ 0.001

Next, we studied the fate of Treg subsets after removal of a short-term polyclonal stimulus. CD4+CD25+ enriched (82 ± 15 % CD4+CD25+ PBL) and CD4+CD25+ depleted (33 ± 5.3 % CD4+CD25+ PBL) cell preparations as well as unseparated PBL from 3 healthy individuals were stimulated with PMA/Ionomycin for 6 h. PMA/Ionomycin was washed out of the cell culture and Treg subsets were analyzed again at 7, 24, 72 and 96 h. In PBL cell cultures, total CD4+CD25+Foxp3+CD127- (7 h vs 72 h, *p* = 0.029) and Helios+IFNγ+ (7 h vs 72 h, *p* = 0.045) Treg increased after removal of the stimulus (Fig. 5a). An increase of CD4+CD25+Foxp3+CD127- (7 h vs 48 h resp. 72 h, *p* = 0.053 resp. *p* = 0.047) was also observed when CD4+CD25- enriched PBL fractions were studied instead of unseparated PBL (Fig. 5c). In contrast, CD4+CD25+ enriched PBL fractions showed only an increase of Helios-IFNγ+ Treg (7 h vs 72 h, *p* = 0.034) (Fig. 5b). Our data do not support the hypothesis [17–24] that removal of the stimulus results in a significant decrease of IFNγ- as well as IFNγ+ CD4+CD25+Foxp3+CD127- Treg due to loss of Foxp3 expression. We found that Helios+ as well as Helios- IFNγ+ Treg increased during the 96 h observation period.

### Foxp3 TSDR DNA methylation status of IFNγ+ and IFNγ- Treg preparations

**Fig. 6:**
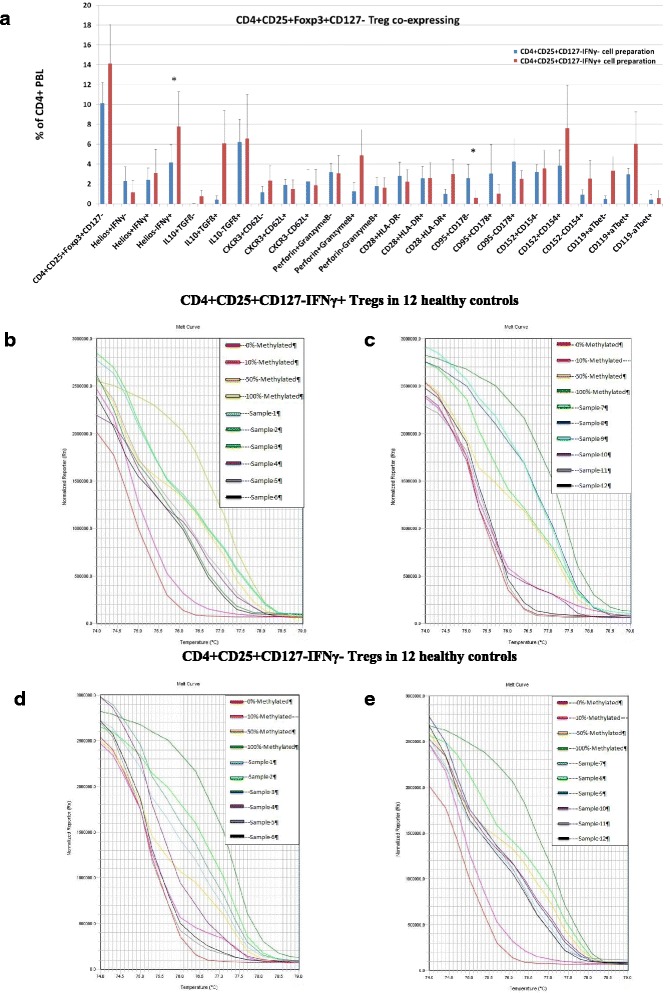
Treg subsets and Foxp3 TSDR DNA methylation analysis in enriched CD4+CD25+CD127-IFNγ+PBL preparations. **a** CD4+CD25+CD127-IFNγ+ Treg were separated from CD4+CD25+CD127-IFNγ- Treg using PBL from 5 different healthy volunteers. CD4+CD25+Foxp3+CD127- co-expressing Helios-IFNγ+ were significantly enriched in the IFNγ+ Treg preparation whereas CD95+CD178- Treg were depleted compared to IFNγ- Treg preparations. Data are given as mean ± SEM. *p ≤ 0.05. **b**-**d** Foxp3 TSDR DNA methylation analysis of enriched CD4+CD25+CD127-IFNγ+ (**b**, **c**) and enriched CD4+CD25+CD127-IFNγ- (**d**, **e**) Treg fractions from 12 healthy individuals (samples 1 – 12). Figure **b** and **d** and Fig. **c** and **e** correspond with each other showing test results of the same individuals. In addition, melt curves of the standards with 0, 10, 50 and 100 % methylated Foxp3 TSDR DNA are depicted in each diagram. 4 of 6 IFNγ+ Treg preparations in Fig. 6b, 3 of 6 IFNγ+ Treg preparations in (**c**), 3 of 6 IFNγ- Treg preparations in (**d**) and 5 of 6 IFNγ- Treg preparations in (**e**) showed a Foxp3 TSDR methylation of <50 % representing mainly demethylated Treg with stable Foxp3 expression

Because IFNγ+ CD4+CD25+Foxp3+CD127- Treg are nearly undetectable in the blood of healthy controls, IFNγ+ Treg were purified by sequential enrichment of CD4+, CD25+, CD127- and IFNγ+ PBL resulting in CD4+CD25+CD127-IFNγ+ and CD4+CD25+CD127-IFNγ- Treg preparations (Fig. 6a). Cell preparations (*n* = 5) enriched of IFNγ+ PBL had higher Helios-IFNγ+ (*p* = 0.043) Treg. Foxp3 TSDR DNA methylation was analyzed in enriched CD4+CD25+CD127-IFNγ+ and CD4+CD25+CD127-IFNγ- Treg preparations from 12 healthy individuals. Figure 6b + c shows the melt curves obtained with CD4+CD25+CD127-IFNγ+ Treg fractions, Fig. 6d + e the melt curves obtained with CD4+CD25+CD127-IFNγ- Treg preparations. 7 of the 12 CD4+CD25+CD127-IFNγ+ and 8 of the 12 CD4+CD25+CD127-IFNγ- Treg preparations showed partial Foxp3 TSDR methylation of <50 % and 3 of the CD4+CD25+CD127-IFNγ+ and 2 of the CD4+CD25+CD127-IFNγ- Treg preparations exhibited a very low Foxp3 TSDR methylation of <10 %, suggesting that IFNγ+ and IFNγ- Treg preparations show similar patterns of demethylated Foxp3 TSDR. It appears that part of IFNγ+ Treg have stable Foxp3 expression and might represent activated nTreg.

## Discussion

We studied frequencies of IFNγ+ Treg subsets in the peripheral blood of healthy individuals and investigated whether IFNγ+ Treg originate from Helios+ CD4+CD25+ thymus-derived nTreg and might represent activated nTreg with Foxp3 TSDR demethylation, stable Foxp3 expression and strong suppressive potency, or, alternatively, differentiate peripherally from Helios- CD4+CD25- conventional T lymphocytes representing aTreg with Foxp3 TSDR methylation, transient expression of Foxp3 and low suppressive capacity [1].

Our data indicate that healthy individuals have both types of IFNγ+ Treg in the blood and that both cell types co-express determinants characteristic for Treg. However, only very few IFNγ+ Treg in the blood can be separated with respect to Helios positivity into activated nTreg and aTreg. Few of IFNγ+ as well as IFNγ- Treg preparations showed strong demethylation of Foxp3 TSDR, supporting the hypothesis that part of IFNγ+ Treg represent activated nTreg. As shown by Hall et al. in rats, activated nTreg form after stimulation with alloantigen in the presence of IFNγ or IL12 and express a Th1-like phenotype [14, 15]. They have a 100- to 1000-fold increased suppressor potency over nTreg that were generated by antigen unspecific stimulation with IL2 alone. These alloantigen-specific IFNγ+ nTreg, formed and activated in the presence of IFNγ, might be suitable candidates for Treg monitoring in organ-grafted patients. An increase of this particular Treg subset might indicate suppression of the graft-specific immune response. In a previous study, we were able to show that renal transplant recipients with good long-term graft function possessed higher proportions of IFNγ+ Treg than patients with impaired long-term graft function [2]. Helios positivity and Foxp3 TSDR methylation status of IFNγ+ Treg were not determined in that study.

Our current in-vitro data show that IFNγ alone is unable to induce this Th1-like Treg subset. However, IFNγ in combination with a second stimulus amplifies the activation of nTreg switching from IFNγ- to IFNγ+, and, in addition, induces the differentiation of IFNγ+ aTreg. This observation supports our hypothesis that IFNγ+ Treg represent the first line of Treg during an immune response. However, what is the fate of IFNγ+ Treg when the immune response is stopped? Do they become apoptotic or do they further differentiate to Th1 lymphocytes, losing their Foxp3 expression and forming potentially harmful graft-specific Th1 lymphocytes? Our data show that during 90 h after elimination of the polyclonal stimulus from the cell culture, both IFNγ+ nTreg and IFNγ+ aTreg continue to increase. Feng et al. reported that in-vitro induced murine IFNγ+ Treg suppress inflammation effectively when transferred to mice with colitis, but differentiate to Th1 lymphocytes when adoptively transferred to mice without inflammation [16]. IFNγ+ Treg that were exposed to an IFNγ-containing milieu during our in-vitro experiments or after transfer into inflamed tissues during the in-vivo experiments of Feng et al. [16] remained phenotypically and functionally stable. Based on the experiments of Feng et al. [16], we speculate that IFNγ+ Treg, when not stimulated further by IFNγ, remain as resting harmless nTreg or Foxp3-IFNγ- Th1 lymphocytes that do not attack the graft in a transplant recipient. We believe that in stable transplant recipients the lack of IL12 and IFNγ in the in-vivo milieu might render these per se graft-specific cells, which differentiated from Th1-like Treg to Th1 lymphocytes, harmless.

As shown in the blood of healthy individuals, Helios+IFNγ+ Treg co-express TGFß but not IL10. Further analysis of Treg phenotypes showed that Treg co-expressed, in-addition, Granzyme B and Perforin as well as Fas (CD95) and FasL (CD178), thereby affording the Treg the capacity to induce lysis and apoptosis of target cells [6]. Moreover, expression of CTLA-4 (CD152) and CD40L (CD154) imply cell-cell contact-dependent immunosuppression by these Treg subsets. CXCR3 and CD62L expression suggests that part of these cells have the potential to enter secondary lymphoid organs as well as inflamed tissues [25, 26]. These Treg exhibit Th1 characteristic properties such as IFNγR1 (CD119) and Tbet expression, which means they have the potency to regulate expression as well as consumption of IFNγ in the cell. CD28 is involved in Treg activation and HLA-DR expression indicates activation of Treg [27]. Our present experiments show that these cell markers are expressed on IFNγ+ as well as IFNγ- Treg in the peripheral blood (frequency of each subset: <10 % of CD4+CD25+Foxp3+CD127- Treg; data not shown) and this observation confirms the results of our previously published in-vitro studies [6, 7]. Expression of TGF, CD183, CD62L, CD152, CD28, CD178, CD95, CD119, T-bet, HLA-DR, Perforin and Granzyme B in/on circulating CD4+CD25+Foxp3+CD127- Treg provides insight into the immunosuppressive armamentarium and the mechanisms of IFNγ+ Treg function. Selection of the most appropriate Treg population for cell therapy is a critical step in ensuring successful clinical outcomes, as reviewed recently [28].

## Conclusions

In the blood of healthy individuals, two-thirds of all CD4+CD25+Foxp3+CD127- Treg were found to be Helios+IFNγ- nTreg. Only a small minority represents Helios+IFNγ+ activated nTreg or Helios-IFNγ+ peripherally induced aTreg. Both IFNγ+ and IFNγ- Treg preparations contain in part strongly demethylated Foxp3 TSDRs, indicating stable Foxp3 expression characteristic for nTreg. During polyclonal stimulation in the presence of rIFNγ, Helios-IFNγ+ aTreg are induced and resting Helios+IFNγ- nTreg differentiate to activated Helios+IFNγ+ thymically-derived nTreg. rIFNγ alone is unable to induce this differentiation. Polyclonal activation induces the expression of IFNγR1 (CD119) and these receptors are necessary for the stimulating effect of auto- and paracrine secreted IFNγ. CD119 is expressed on IFNγ+, IFNγ-, Helios+ as well as Helios- Treg. The frequency of each subset in the peripheral blood was <5 % of CD4+CD25+Foxp3+CD127- Treg (data not shown). The present data provide further insight into the in-vivo and in-vitro characteristics of IFNγ+ Treg and help to understand their role in immunoregulation. Alloantigen-specific demethylated IFNγ+ Helios+ nTreg might represent a suitable marker for monitoring graft-specific immunosuppression in renal transplant recipients.

## Methods

### Healthy controls

Laboratory staff served as healthy controls. All controls gave informed consent for the tests performed within this study. The study was reviewed by the ethics committee of the University of Heidelberg and was performed in accordance with the ethical standards laid down in the 2000 Declaration of Helsinki as well as the Declaration of Istanbul 2008. All healthy individuals gave their informed consent prior to their inclusion in the study.

### Stimulation of PBL using PMA/Ionomycin

PBL were separated from heparinized or EDTA whole blood by Ficoll densitiy gradient centrifugation and stimulated for different time intervals using a mixture of phorbol 12-myristate 13-acetate (PMA; final concentration in medium: 10 ng/ml; Sigma Aldrich, Munich, Germany) and ionomycin (1 μg/ml; Sigma Aldrich, Munich, Germany) in RPMI medium containing 10 % FCS, L-Glutamin, and Penicillin/ Streptomycin (all from Invitrogen Gibco, Paisley, Scotland) as described previously [5].

### Determination of different PBL subsets

PBL subsets were determined as described previously [2, 5]. For analysis of determinants on the cell surface, PBL were incubated with fluorochrome-labelled monoclonal antibodies against CD3, CD4, CD25, CD28, CD62L, CD95, CD119, CD127, CD152, CD154, CD178, CD252, HLA-DR, and CD183 (CXCR3) (all from BD Biosciences). Intracellular determinants were stained with fluorochrome-labelled monoclonal antibodies against Foxp3, IFNγ (clone B27), IL4, IL10, Granzyme B, Perforin, T-bet (all from BD Biosciences), Helios (ebioscience, Frankfurt, Germany) and TGFß_1_ (R&D systems, Wiesbaden). Briefly, PBL were incubated with combinations of monoclonal antibodies for 30 min as described and eight-color fluorescence was analyzed using a FACSCanto II triple-laser flow cytometer (BD Biosciences) [2, 5]. When, in addition, intracellular proteins were studied, cell membranes were permeabilized using BD Perm/Wash buffer (BD Biosciences). At least 100,000 events were analyzed in the initial FSC/SSC dot plot. IFNγ monoclonal antibody used for cell separation (BD clone 4S.B3) and IFNγ monoclonal antibody used for cell staining (BD clone B27) were not competitive (data not shown).

### Enrichment of CD4+CD25+CD127-IFNγ+ Treg

Using this procedure, CD4+CD25+CD127-IFNγ+ Treg were enriched by >80 %. CD4^+^ and CD25+ PBL were subsequently separated by positive selection using Strep-Tactin magnetic Microbeads (IBA, Göttingen, Germany) according to the instructions of the manufacturer. Briefly, 4 μl Fab-Strep were mixed with 1 μl Buffer IS. Then, 15 μl from the homogeneously resuspended Strep-Tactin Magnetic Microbeads were added to the Fab-Strep solution and incubated overnight at 4 °C. The pre-incubated Fab-Strep Microbead preparation was added to the cells and mixed gently for 20 min at 4 °C. Then, 5 ml of buffer IS were added to the cell/bead preparation. Magnetically labelled cells were separated by placing the tube for 3 min onto the StrepMan magnet. Negative cell fraction was removed with the supernatant whereas the positive cell fraction was flushed off the tube by resuspension in 5 ml buffer IS. Magnetic selection was repeated twice. Labelled cells were resuspended in 10 ml of a 1 mM D-biotin working solution and incubated for 10 min at room temperature. Then, tube was placed on the StrepMan magnet for 3 min and supernatant containing the target cell fraction was pipetted off the tube and transferred to another tube. The last step was repeated twice. Cells were centrifuged by 400 g for 10 min and resuspended in buffer. Using this procedure, CD4+ and CD25+ PBL were enriched. Then, CD127- PBL were separated by negative selection and IFNγ+ PBL were separated by subsequent positive selection, as described previously [7]. First, cells were incubated with CD127 monoclonal antibody (BD Biosciences, Heidelberg, Germany). Then, CD4^+^CD25^+^CD127^−^ were separated from CD4^+^CD25^+^CD127^−^ using streptavidin-coupled beads (Dynabeads Biotin Binder, Invitrogen, Dynal Oslo, Norway) according to the instructions of the manufacturer. Procedure was repeated using IFNγ monoclonal antibody (BD Biosciences, Heidelberg, Germany, IFNγ clone 4S.B3). Separated PBL were added to cell cultures.

### DNA isolation

Genomic DNA of separated CD4+CD25+CD127-INFγ+ and CD4+CD25+CD127-INFγ- T cells was extracted using the QIAamp cultured cells Mini Kit (Qiagen, Hilden, Germany) according to the protocol of the manufacturer. DNA was stored in Eppendorf tubes (Eppendorf, Hamburg, Germany) at −20 °C.

### Bisulfite conversion of genomic DNA

Bisulfite converts unmethylated cytosines to uracil, whereas methylated cytosines remain unreactive. After conversion of unmethylated cytosines to uracil by bisulfite treatment and subsequent PCR-mediated conversion of uracils to thymine, methylated and unmethylated alleles are predicted to differ in thermal stability because of their different CpG contents. Bisulfite conversion of genomic DNA was performed using the EZ DNA Methylation Kit (Zymo research, Freiburg, Germany) according to the manufacturer’s instructions. CT Conversion Reagent powder was solved with 750 μl water and 210 μl M-Dilution buffer. 5 μl of M-Dilution buffer was added to the isolated DNA samples. Total volume was adjusted to 50 μl with water and mixed. CT Conversion Reagent and DNA samples were incubated separately in the dark at 37 °C for 15 min. After incubation, 100 μl of CT Conversion Reagent was added to each DNA sample. Samples were mixed and incubated in the dark at 50 °C for 12–16 h. After incubation DNA samples were placed on ice for at least 10 min. Then, DNA samples were loaded into a Zymo-Spin IC Column containing 400 μl M-Binding buffer, mixed, and centrifuged at 10 000 g for 30 s. Additional 100 μl of M-Wash buffer were added to the column and centrifuged at 10 000 g for 30 s. Then, another 200 μl of M-Desulphonation buffer were added to the column, incubated at room temperature for 20 min and, finally, centrifuged at 10 000 g for 30 s. Further, 200 μl of M-Wash buffer were added to the column and centrifuged at 10 000 g for 30 s. The procedure was repeated. Thereafter, the Zymo-Spin IC Column was placed into a clean 1.5 ml microcentrifuge tube (Eppendorf), 15 μl of M-Elution buffer were added to the column matrix, and samples were centrifuged at 10 000 g for 30 s. DNA was eluted in Eppendorf tubes and stored at −20 °C for later use.

### Foxp3 TSDR DNA methylation analysis

HRM analysis after bisulfite treatment identifies methylation variations in the Foxp3 gene. Bisulfite modified DNA was subjected to PCR according to the protocol for HRM analysis provided by the primer manufacturer (EpigenDx, Hopkinton, MA, USA). ADS3576 primers were used for the amplification of the promoter and 5’UTR region, and ADS783 primers for the amplification of the intron 1 TSDR of the Foxp3 gene. PCR was carried out in a 21 μl total volume containing: 2.1 μl PCR buffer, 0.27 μl MgCl_2_, 0.42 μl dNTPs, 1.05 μl SYBR Green dye (EpigenDx), 1.26 μl each of primers ADS783 and ADS3576 (Human Foxp3 Methylation Panel, EpigenDx), 0.12 μl Hot Start Taq polymerase (Qiagen), and 1.5 μl of bisulfite-treated genomic DNA (concentration 5 ng/μl). The amplification protocol with ADS783 primers consisted of incubation at 95 °C for 15 min followed by 45 cycles at 95 °C for 30 s, 62 °C for 30 s and 72 °C for 30 s with a final extension step at 72 °C for 5 min. The amplification protocol with ADS3576 primer consisted of an incubation at 95 °C for 15 min followed by 45 cycles at 95 °C for 30 s, 59 °C for 30 s and 72 °C for 30 s with a final extension step at 72 °C for 5 min. Melting was performed from 60 to 90 °C at a melt rate of 1 %. Each sample was analyzed in triplicate. DNA methylation analysis and diagram generation were performed using real-time PCR software (Applied Biosystems 7500, Foster City, CA, USA).

### Standard curve

7 commercially available standards with different proportions of methylated Foxp3 template DNA were used to estimate methylation status of the samples (0, 5, 10, 25, 50, 75, and 100 %) (Human Methylation Controls – Mix, EpigenDx). Templates were pretreated with bisulfite. For clarity, only 4 standards are depicted in the figures.

### Statistics

All assays were repeated at least 3 times with PBL of different healthy controls. Representative test results and/or mean ± SEM were depicted in the figures. For statistical analysis PASW Statistics program version 21 (IBM, Chicago, Illinois, USA) and Wilcoxon (Figs. 3, 4 and 6a) and Student t test (Fig. 5) were used. P-values ≤0.05 were considered significant. P-values of 0.056 were considered to show a trend.

## Abbreviations

aTreg: Adaptive T regulator cell
Fab: Fragment antigen binding
HRM: High resolution melt
IL: Interleukin
MLC: Mixed lymphocyte culture
nTreg: Natural T regulator cell
PBL: Peripheral blood lymphocytes
PCR: Polymerase chain reaction
PMA: Phorbol 12-myristate 13-acetate
rIFNγ: Recombinant interferon gamma
Tbet: T-box expressed in T cells
TCR: T cell receptor
Th1: T helper type 1
TSDR: Treg-specific demethylated region
